# Trimester-specific reference intervals and profile of coagulation parameters for Chinese pregnant women with diverse demographics and obstetric history: a cross-sectional study

**DOI:** 10.1186/s12884-023-05571-z

**Published:** 2023-06-06

**Authors:** Jing Dai, Peimin Mao, Cunying Pu, Xuefeng Wang, Xiaoyan Liu

**Affiliations:** 1 Department of Laboratory Medicine, Shanghai Jiaotong University School of Medicin Ruijin Hospital, Shanghai, China; 2 Department of Blood Transfusion, Obstetrics and Gynecology Hospital of Fudan University, Shanghai, China; 3 Roche Diagnostics (Shanghai) Limited, Medical and Scientific Affairs, Minhang District, Shanghai, China

**Keywords:** Coagulation, Reference intervals, Trimester, Advanced maternal age, Obstetric history, Parity, Gravidity

## Abstract

**Background:**

Owing to the changes in childbirth policy in China, this work aimed to update the trimester-specific reference intervals (RIs) for Chinese pregnant women with diverse demographics and obstetric history. This study also investigated how advanced maternal age (AMA) (> 35 years old), gravity, and parity influence gestational coagulation parameters.

**Methods:**

In this prospective cross-sectional study, five coagulation parameters were measured using assays provided by Roche diagnostics on Cobas t 711: prothrombin time (PT), activated partial thromboplastin time (APTT), thrombin time (TT), fibrinogen (Fib), and D-dimer, and the trimester-specific 2.5th -97.5th and 95th (D-dimer only) percentiles RIs were established accordingly. Linear regressions were undertaken to analyze the association with demographic characteristics and obstetric history for each parameter.

**Results:**

893 eligible pregnant women in different trimesters and at AMA/non-AMA and 275 non-pregnant healthy women were enrolled. For the first, second, and third trimester, respectively, RIs were as follows: APTT (s): 24.8–35.7, 24.6–34.1, and 23.5–34.7; TT (s): 14.4–17.3, 14.1–16.7, and 14.2–17.5; PT (s): 8.30–10.20, 8.00-9.77, and 7.92–9.57; PT-INR: 0.86–1.06, 0.83–1.02, and 0.82–0.98; Fib (g/L): 2.76–4.97, 3.14–5.31, and 3.44–5.93; D-dimer (µg/ml): 0-0.969, 0-2.14, and 0-3.28. No statistically significant differences were observed in TT, D-dimer, and APTT between the AMA and non-AMA women, while PT and PT-INR were shorter and Fib was higher in the AMA group. The association of gravidity and parity with each coagulation parameter is statistically significant (p < 0.05). PT and PT-INR were shortened and D-dimer decreased as gravidity increased. Longer PT and PT-INR, shorter APPT, higher D-Dimer, and lower Fib were associated with increasing parity.

**Conclusions:**

This work updated the gestational coagulation profiles of Chinese pregnant women and established trimester-specific RIs accordingly. Establishing specific RIs based on AMA, parity, and gravidity might not be necessary.

**Supplementary Information:**

The online version contains supplementary material available at 10.1186/s12884-023-05571-z.

## Background

During pregnancy, changes occur in many aspects of hemostasis, which result in a hypercoagulable state [1]. Hypercoagulability can be life-threatening and increase the risk of pregnancy-related venous thromboembolism (VTE) [2]. Therefore, monitoring the coagulation status is crucial to optimizing timely interventions, and several coagulation parameters: prothrombin time (PT), activated partial thromboplastin time (APTT), thrombin time (TT), fibrinogen (Fib), and D-dimer, have been actively used in the monitoring. As the conventional reference intervals (RIs) for non-pregnant populations are usually not adaptable for pregnant women, trimester-specific reference intervals were established and recognized for accurately diagnosing hemostatic status during pregnancy [1, 3].

Although multiple studies on the trimester-specific RIs and profile of coagulation parameters of Chinese pregnant women have been reported [4–7], the study that enrolled most patients was published ten years ago (2012) [7]. This work initially aims to update trimester-specific reference intervals that might fulfill the latest clinical needs towards managing pregnant women in light of the two-child implementation (2015) and the recent raising of the three-child (2021) policy in China.

Due to the changes in childbirth policy, the population at advanced maternal age (AMA) and with a more diverse obstetric history, including multigravida and multiparous, could increase in China [8]. AMA generally describes pregnant women over 35 years old and has gained significant attention due to the increased risk for multiple complications [9]. Recent studies suggested that the association of AMA with adverse obstetrical outcomes also exists in Chinese pregnant women [10–14]. However, only a few studies have examined how AMA and obstetric history may affect their coagulation profiles. The secondary objective of this study is to look into the potential relationship between the five coagulation parameters and AMA, parity, and gravidity, considering the insufficiency of these studies.

## Methods

### Study design

**Fig. 1:**
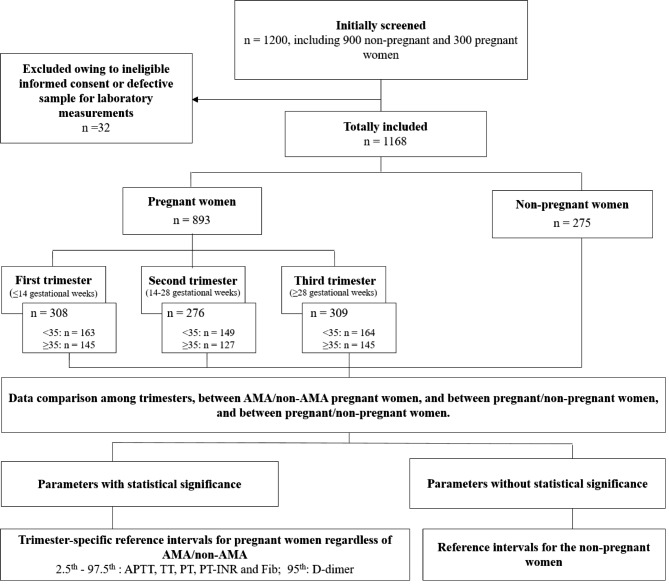
Study flowchart

Figure 1 shows the flowchart of this prospective cross-sectional study. This study was designed to screen 1200 subjects who meet the eligibility criteria listed below, including 300 healthy non-pregnant women and 900 pregnant women. From May 2021 to January 2022, 900 pregnant women who attended routine prenatal care at the outpatient of Obstetrics and Gynecology Hospital of Fudan University were recruited. From May 2021 to November 2021, 300 healthy non-pregnant women who attended routine health care or physical examination at the outpatient of Ruijin Hospital, Shanghai Jiao Tong University School of Medicine, were recruited to compose the control group. Gravidity is defined as the number of times a woman has been pregnant. Parity is defined as the number of times a woman has given birth to a fetus with a gestational age of 24 weeks or more, regardless of pregnancy outcomes.

### Eligibility criteria

For the non-pregnant health women, the inclusion criteria were (1) women at reproductive age (18–50 years old); (2) healthy weight state with BMI within the range of 18.5–24.9; (3) normal heart, liver, abnormal blood coagulation, and blood system diseases; Exclusion criteria were: (1) hypertension, diabetes, thyroid disease, abnormal blood coagulation, and blood system diseases; (2) current on drugs that may influence coagulation and fibrinolysis, including but not limited to acetylsalicylic acid, oral anticoagulants, phenprocoumon warfarin, heparin, antiplatelet drugs and contraceptive; (3) history of infection, fever, and surgery in the past 30 days; (4) personal or family history of bleeding and clotting disorders, cardiovascular, cerebrovascular, liver, kidney, and autoimmune diseases.

For the pregnant women, included subjects were: (1) with natural singleton pregnancy; (2) among 18–50 years old; (3) with pre-pregnancy baseline BMI within the range of 18.5 ~ 24.9. Pregnant women with any of the following conditions that may cause abnormal blood coagulation will be excluded: (1) hypertension, diabetes, abnormal thyroid function; (2) prenatal bleeding due to either placental abruption or placenta pevia; (3) long-term bed-rest prevention and cervical insufficiency; (4) coagulation disorder, hematological disease, or the current using of drugs that may influence coagulation and fibrinolysis. The drugs included but were not limited to acetylsalicylic acid, oral anticoagulants, phenprocoumon, warfarin, heparin, and antiplatelet drugs; (5) history of infection, fever, and surgery in the past 30 days; (6) history of abnormal pregnancy or abortion; (7) personal or family history of bleeding and clotting disorders, cardiovascular, cerebrovascular, liver, kidney, and autoimmune diseases.

### Laboratory measurements

Each sample was collected and placed in 3.2% sodium citrate anticoagulant tubes with at least 1.0 mL volume. Samples that may yield incorrect results were excluded, including those that were clotted, hemolyzed, lipemic, or icteric. Each included sample was strictly handled following a 9:1 blood: anticoagulant ratio within one hour after collection, and the plasma was separated by centrifugation at 2500 g for 15 min to achieve a platelet count of less than 10,000/µL. Coagulation parameters for each plasma sample were analyzed using commercial reagents on a Cobas t 711 coagulation analyzer (Roche Diagnostics GmbH) within 2 h after centrifugation. The reagents used for each coagulation parameter were: Cobas aPTT screen clotting time assay for APTT, Cobas PT Rec clotting time assay for PT, Cobas TT for TT, Cobas Fibrinogen clauss clotting assay for Fib, and D-DI2 (or Tina-quant® D-Dimer Gen. 2) particle-enhanced immunoturbidimetric assay for D-dimer. All the reagents and instruments were used strictly according to the standard operating procedure (SOP) and package inserts provided by the manufacturer.

### Statistical analysis

The reference ranges were calculated based on the distribution of PT, APTT, TT, Fib, and D-dimer levels. The normality of the data was assessed using the D’Agostino-Pearson test. The non-parametric 2.5th-97.5th and 95th (D-dimer) percentiles RIs of the coagulation parameters were established. Baseline characteristics were described as mean (standard deviation, SD) and median (interquartile range, IQR) for continuous variables and frequency (%) for categorical variables. T-test and ANOVA were used to test the difference between normally distributed continuous variables between two and three groups. Mann-Whitney U and Kruskal-Wallis H tests were used to test the difference between skew-distributed continuous variables between two and three groups. Mantel-Hazel chi-square tests were used to test the difference between categorical variables. Univariate and multivariate linear regression analyses were undertaken to test the association of the level of coagulation factors with age, gestational weeks, gravidity, and parity. Afterward, log transformations were made for the dependents to ensure a normally distributed residual. All statistical analyses were performed using R 4.0.0 software. P ≤ 0.05 was considered statistically significant.

## Results

### Baseline demographics, coagulation characteristics, and obstetric history

In this study, 1168 subjects were eventually included, with 32 subjects excluded due to ineligible informed consent or defective sample for laboratory measurements (Fig. 1). A total of 893 pregnant women were included, including 308, 276, and 309 in the first, second, and third trimester, respectively. Their baseline demographics, coagulation characteristics, and obstetric history in each trimester were summarized in Table 1. Compared with non-pregnant women, the pregnant women have a significantly shorter APTT, PT, PT-INR, and TT as well as higher D-dimer and Fib with p < 0.01 (Table S1). No statistically significant difference in age, baseline BMI (BMI before pregnancy), and the number of pregnant women with certain gravidity or parity among the trimesters were determined. Significant differences in current BMI and the five coagulation parameters (p < 0.001) were identified among the trimesters that current BMI, D-dimer, and Fib increased throughout pregnancy, with the highest value at the third trimester. On the contrary, TT significantly decreased throughout the pregnancy, with the shortest time period in the first trimester.

**Table 1:** Demographics, coagulation characteristics, and obstetric history of pregnant women in different trimesters

		First Trimester	Second trimester	Third trimester	p
Age	Median (IQR)	34.000 (29.000–37.000)	33.000 (29.000–37.000)	34.000 (29.000–36.000)	0.893
	Mean (SD)	33.026 (4.545)	33.163 (4.543)	32.981 (4.485)	0.881
Baseline BMI	Median (IQR)	21.039 (19.721–22.308)	20.831 (19.719–22.420)	21.025 (19.706–22.481)	
	Mean (SD)	21.099 (1.678)	21.134 (1.708)	21.149 (1.666)	0.930
Current BMI	Median (IQR)	21.554 (20.278–22.931)	23.729 (22.343–25.450)	25.771 (24.205–27.401)	
	Mean (SD)	21.785 (1.929)	23.866 (2.364)	25.882 (2.226)	< 0.001
APTT(s)	Median (IQR)	29.600 (27.600–31.300)	28.200 (26.700-29.925)	28.600 (27.000-30.400)	< 0.001
	Mean (SD)	29.690 (2.904)	28.454 (2.454)	28.822 (2.509)	
TT (s)	Median (IQR)	15.600 (15.200–16.000)	15.200 (14.900–15.800)	15.100 (14.700–15.600)	< 0.001
	Mean (SD)	15.637 (0.688)	15.337 (0.769)	15.319 (0.988)	
PT	Median (IQR)	9.070 (8.760–9.350)	8.590 (8.370–8.870)	8.580 (8.310–8.870)	< 0.001
	Mean (SD)	9.088 (0.466)	8.667 (0.459)	8.628 (0.422)	
PT-INR	Median (IQR)	0.940 (0.911–0.969)	0.899 (0.876–0.926)	0.889 (0.861–0.917)	< 0.001
	Mean (SD)	0.942 (0.048)	0.906 (0.048)	0.892 (0.043)	
Fib (g/L)	Median (IQR)	3.630 (3.290–3.970)	4.080 (3.768–4.460)	4.500 (4.150–4.920)	< 0.001
	Mean (SD)	3.671 (0.550)	4.131 (0.550)	4.553 (0.604)	
D-dimer (µg/ml)	Median (IQR)	0.308 (0.188–0.451)	0.809 (0.547–1.162)	1.100 (0.808-1.600)	< 0.001
	Mean (SD)	0.397 (0.351)	0.955 (0.575)	1.408 (1.033)	
No. of pregnant women with certain gravidity (%)	1	143 (46.4)	129 (46.7)	148 (47.9)	0.578
	2	90 (29.2)	68 (24.6)	90 (29.1)	
	3	42 (13.6)	46 (16.7)	35 (11.3)	
	> 3	33 (10.7)	33 (12.0)	36 (11.7)	
No. of pregnant women with certain parity (%)	0	191 (62.0)	176 (63.8)	211 (68.3)	0.340
	1	109 (35.4)	92 (33.3)	95 (30.7)	
	2	8 (2.6)	7 (2.5)	3 (1.0)	
	3	0 (0.0)	1 (0.4)	0 (0.0)	

*Note*: Ages were normally distributed, and one-way ANOVA was used to test the difference in age among trimesters; BMI and all the coagulation parameters were skewed distributed, and the Kruskal-Wallis H test was used to test the difference among groups; Differences in categorical data: No. of pregnant women with certain gravidity and parity were tested using the Mantel-Hazel chi-square test

Afterward, we divided the pregnant women based on the definition of AMA, regardless of trimester. There were 476 pregnant women in the regular maternal age group (< 35 years old), and 417 in the AMA group. Table S2 shows that PT and APTT were significantly longer in the regular maternal age group, while other demographic and coagulation differences between the two groups were not statistically significant. The number of pregnant women with certain obstetric history between the two age groups was significant (p < 0.001). AMA pregnant women tended to have multigravida (gravidity > 1) and multiparous (parity > 0).

275 non-pregnant healthy women were enrolled as the control group, their baseline demographic and coagulation characteristics and the subgroups stratified by gender or age are summarized in Table S2. Comparing the coagulation parameters in the two age groups, no statistically significant differences were observed in TT, D-dimer, and APTT, while PT and PT-INR were shorter and Fib was higher in the elder group (> 35 years old).

### Reference intervals of coagulation parameters

**Fig. 2:**
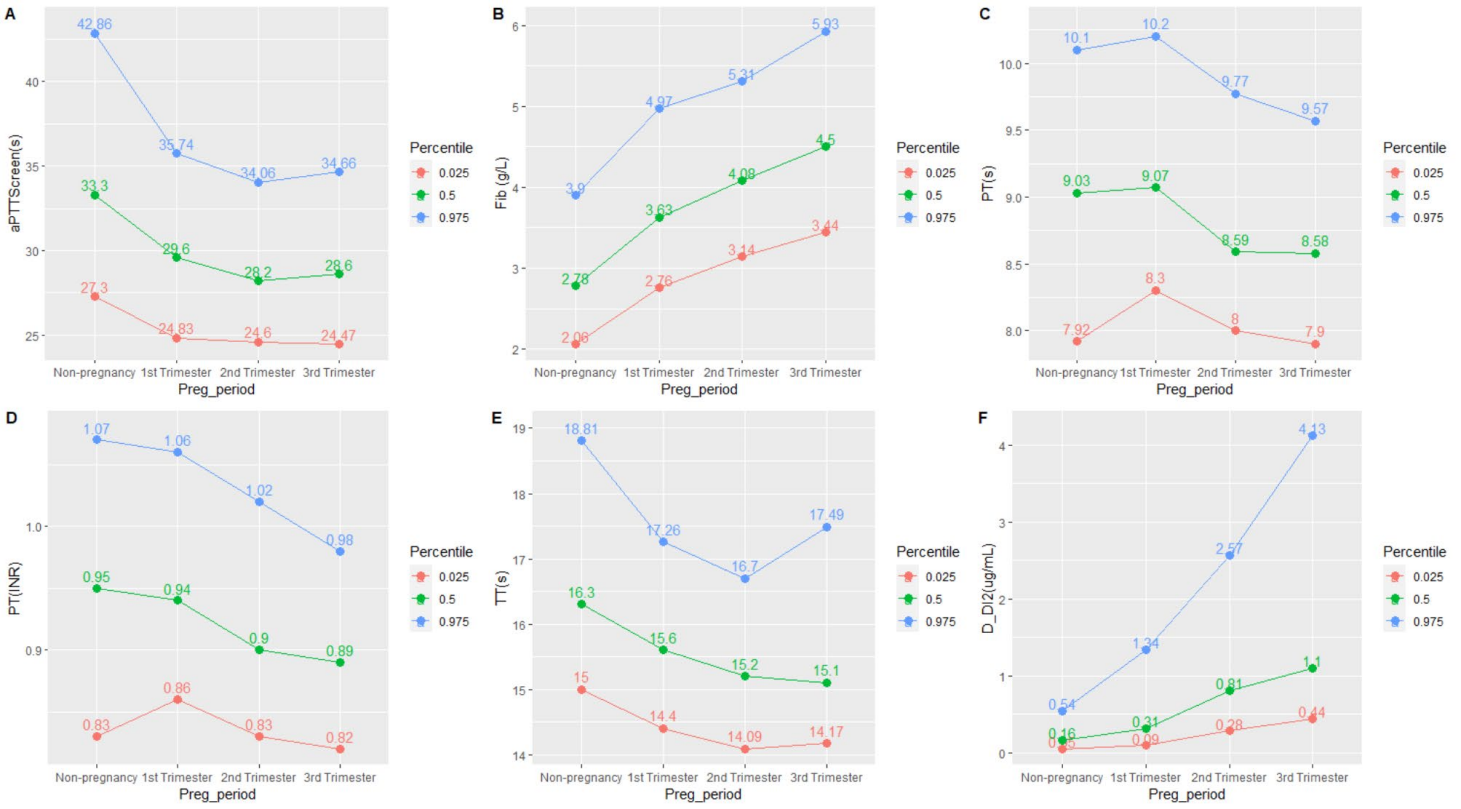
Percentile curves of coagulation parameters for pregnant and non-pregnant women in different trimesters. **A**: APPT; **B**: Fib; **C**:PT; **D**: PT-INR; **E**: TT; **F**: D-dimer

We established the RIs of five coagulation parameters for healthy pregnant and non-pregnant women (Table 2). Variation of coagulation parameters among trimesters is demonstrated in Fig. 2. Overall, the third trimester showed increased Fib and D-dimer compared with non-pregnant women and earlier trimesters. The first trimester showed decreased PT-INR and PT compared with later trimesters.

**Table 2:** Coagulation reference intervals of non-pregnant and pregnant women

	Non-pregnant women	Pregnant women			
		Total	First trimester	Second trimester	Third trimester
**APTT(s)**	27.5–43.1	24.6–34.9	24.8–35.7	24.6–34.1	23.5–34.7
**TT(s)**	14.7–18.6	14.3–17.1	14.4–17.3	14.1–16.7	14.2–17.5
**PT(s)**	7.96–10.30	7.92–10.1	8.30–10.20	8.00-9.77	7.92–9.57
**PT-INR**	0.83–1.08	0.83–1.03	0.86–1.06	0.83–1.02	0.82–0.98
**Fib (g/L)**	2.00-3.89	2.89–5.47	2.76–4.97	3.14–5.31	3.44–5.93
**D-dimer (µg/ml)**	< 0.39	< 2.434	< 0.969	< 2.14	< 3.28

The longest APTT presents in the non-pregnant women (27.3–42.9 s), and the variation of this parameter among the three trimesters is slight (Fig. 2A). Fib gradually increased before and throughout the pregnancy and reached the highest level in the third trimester (3.44-5.93 g/L, Fig. 2B); For PT and PT-INR, they become shorter with the increase of trimester, and the longest duration is observed in the first trimester (PT: 8.30–10.20 s, Fig. 2C; PT-INR: 0.83–1.03 (Fig. 2D). TT shortens in early pregnancy (first and second trimester), its variance increased in the third trimester (Fig. 2E); D-dimer progressively increased before and throughout the pregnancy and reached the highest level in the third trimester (3.28 µg/ml) (Fig. 2F).

### Exploring the underlying factors affecting gestational coagulation parameter levels

Univariate and multivariate linear regression analyses were undertaken to explore the association of gestational level with several parameters (Table 3). A negative multivariable coefficient revealed a negative association and vice versa.

**Table 3:** Univariate and multivariate linear regression analysis of each gestational coagulation parameter

	Number	Adjusted coefficient (univariate)	Adjusted coefficient (multivariate)
**Dependent: APTT (s)**			
Age		-0.003 (-0.005 to -0.001, p = 0.001)	-0.003 (-0.005 to -0.001, p = 0.004)
Gestational weeks		-0.002 (-0.003 to -0.001, p < 0.001)	-0.002 (-0.003 to -0.001, p < 0.001)
Gravidity	1	-*	-
	2	-0.015 (-0.036 to 0.006, p = 0.154)	-0.004 (-0.029 to 0.021, p = 0.735)
	3	-0.008 (-0.034 to 0.018, p = 0.552)	0.007 (-0.026 to 0.040, p = 0.669)
	≥ 3	-0.021 (-0.050 to 0.007, p = 0.145)	0.007 (-0.031 to 0.045, p = 0.729)
Parity	0	-	-
	1	-0.014 (-0.032 to 0.005, p = 0.150)	-0.004 (-0.031 to 0.022, p = 0.753)
	2	-0.017 (-0.079 to 0.044, p = 0.579)	-0.016 (-0.083 to 0.052, p = 0.648)
**Dependent: D-dimer**			
Age		0.010 (-0.008 to 0.027, p = 0.265)	0.014 (-0.000 to 0.028, p = 0.058)
Gestational weeks		0.081 (0.075 to 0.086, p < 0.001)	0.081 (0.075 to 0.086, p < 0.001)
Gravidity	1	-	-
	2	0.029 (-0.160 to 0.217, p = 0.764)	-0.123 (-0.286 to 0.041, p = 0.141)
	3	-0.131 (-0.372 to 0.110, p = 0.287)	-0.259 (-0.473 to -0.045, p = 0.017)
	≥ 3	-0.061 (-0.321 to 0.198, p = 0.642)	-0.298 (-0.547 to -0.049, p = 0.019)
Parity	0	-	-
	1	0.001 (-0.168 to 0.169, p = 0.995)	0.201 (0.028 to 0.374, p = 0.023)
	2	-0.422 (-0.985 to 0.140, p = 0.141)	0.067 (-0.374 to 0.508, p = 0.764)
**Dependent: Fib (g/L)**			
Age		0.002 (-0.001 to 0.006, p = 0.241)	0.006 (0.002 to 0.009, p = 0.001)
Gestational weeks		0.013 (0.011 to 0.014, p < 0.001)	0.012 (0.011 to 0.014, p < 0.001)
Gravidity	1	-	-
	2	-0.050 (-0.087 to -0.012, p = 0.009)	-0.020 (-0.057 to 0.018, p = 0.300)
	3	-0.045 (-0.093 to 0.003, p = 0.066)	0.002 (-0.047 to 0.051, p = 0.946)
	≥ 3	-0.019 (-0.070 to 0.033, p = 0.473)	0.002 (-0.047 to 0.051, p = 0.946)
Parity	0	-	-
	1	-0.078 (-0.111 to -0.044, p < 0.001)	-0.083 (-0.123 to -0.043, p < 0.001)
	2	-0.021 (-0.132 to 0.090, p = 0.706)	-0.015 (-0.116 to 0.086, p = 0.771)
**Dependent: PT (s)**			
Age		0.000 (-0.001 to 0.001, p = 0.642)	0.000 (-0.002 to 0.001, p = 0.440)
Gestational weeks		-0.003 (-0.004 to -0.003, p < 0.001)	0.003 (-0.004 to -0.003, p < 0.001)
Gravidity	1	-	-
	2	-0.006 (-0.019 to 0.007, p = 0.344)	-0.015(-0.029 to -0.001, p = 0.040)
	3	-0.006 (-0.011 to 0.022, p = 0.493)	-0.010 (-0.028 to 0.009, p = 0.307)
	≥ 3	-0.006 (-0.024 to 0.011, p = 0.475)	-0.023 (-0.044 to -0.002, p = 0.035)
Parity	0	-	-
	1	0.009 (-0.003 to 0.020, p = 0.133)	0.018 (0.003 to 0.033, p = 0.016)
	2	0.043 (0.006 to 0.081, p = 0.025)	0.049 (0.011 to 0.087, p = 0.011)
**Dependent: PT-INR**			
Age		0.002 (0.001 to 0.003, p = 0.003)	0.002 (0.000 to 0.003, p = 0.007)
Gestational weeks		-0.003 (-0.004 to -0.003, p < 0.001)	-0.003 (-0.004 to -0.003, p < 0.001)
Gravidity	1	-	-
	2	0.002 (-0.010 to 0.015, p = 0.725)	-0.011 (-0.024 to 0.003, p = 0.120)
	3	0.014 (-0.001 to 0.030, p = 0.074)	-0.008 (-0.026 to 0.009, p = 0.348)
	≥ 3	0.001 (-0.016 to 0.018, p = 0.881)	-0.029 (-0.049 to -0.008, p = 0.006)
Parity	0	-	-
	1	0.015 (0.004 to 0.026, p = 0.006)	0.016 (0.002 to 0.031, p = 0.026)
	2	0.060 (0.023 to 0.097, p = 0.001)	0.060 (0.024 to 0.097, p = 0.001)
**Dependent: TT (s)**			
Age		0.001 (0.000 to 0.002, p = 0.018)	0.001 (-0.000 to 0.002, p = 0.129)
Gestational weeks		-0.001 (-0.002 to -0.001, p < 0.001)	-0.001 (-0.002 to -0.001, p < 0.001)
Gravidity	1	-	-
	2	0.013 (0.001 to 0.025, p = 0.030)	0.011 (-0.003 to 0.025, p = 0.136)
	3	0.005 (-0.010 to 0.020, p = 0.548)	0.001 (-0.019 to 0.017, p = 0.914)
	≥ 3	0.016 (-0.000 to 0.032, p = 0.053)	0.006 (-0.016 to 0.027, p = 0.614)
Parity	0	-	-
	1	0.01 (-0.00 to 0.02, p = 0.090)	-0.00 (-0.02 to 0.01, p = 0.960)
	2	0.04 (0.00 to 0.07, p = 0.034)	0.03 (-0.01 to 0.06, p = 0.175)

* Reference group

In all multivariable analyses, age as an independent variable showed a statistically significant association (p < 0.05) that owed small coefficients with APTT (-0.003), PT-INR (0.002), and Fib (0.006). The results of the multivariable analysis on gestational weeks are comparable to the association of coagulation parameters with trimesters shown in Fig. 2. APTT, TT, PT, and PT-INR shorten as gestational weeks increase (p < 0.001), while D-dimer and Fib levels significantly increase (p < 0.001).

For gravidity and parity, their association with every coagulation parameter is statistically significant (p < 0.05). Gravidity 1 and parity 0 were set as the reference to observe how the increase of gravidity and parity affected the changing of parameters. With the increase in gravidity, PT and PT-INR were shortened, while D-dimer decreased. Longer PT and PT-INR, shorter APPT, higher D-Dimer, and lower Fib were associated with increasing parity. Parity 1 was associated with an increase in D-dimer [multivariate adjusted coefficient: 0.201 (0.028 to 0.374, p = 0.023)] while a decrease in Fib [multivariate adjusted coefficient: -0.083 (-0.123 to -0.043, p < 0.001)]. The increase of PT and PT-INR at parity 1 and 2 from parity 0 is significant (p < 0.05).

## Discussions

### Gestational coagulation profiles and reference intervals of chinese pregnant women

This work identified statistically significant differences in each parameter between non-pregnant and pregnant women (p < 0.001) and established RIs for pregnant women. Comparing the pregnant to non-pregnant women, the shorter APTT, PT, and TT revealed an increase in clotting activity, and the higher D-dimer and Fib levels suggested the growth of localized fibrinolytic activity on the placental uterine interface [15]. It should be noted that while the coagulation reference intervals were established for non-pregnant women (healthy women) in this study, their application scenario might be limited because the unnecessary established of gender-specific coagulation reference intervals is a well-recognized viewpoint in clinical laboratories that has been recently further approved in a study of us [16]. For the actual needs of clinical analysis and diagnosis, we also establish the coagulation reference intervals for healthy Chinese population by combing the coagulation parameters of 267 healthy adult men recruited in the previous study with parameters of the non-pregnant women obtained in this study, see Table S3 in the supplementary material.

Several clotting factors change during pregnancy: XIII, XII, X, VIII, VWF, FVII, and Fib increase and peak in the third trimester; FII, FV, and FIX slightly increase or remain unchanged; D-dimer increases, indicating increased fibrinolysis following fibrin formation; FXI is the only blood coagulation factor that decreases [1]. These changes necessitated understanding the changing of coagulation parameters with trimesters and gestational weeks in Chinese pregnant women, while the study that enrolled most patients was published ten years ago (2012) [5]. Thus, we consider that the RIs should be updated based on a recent cohort.

In this study, the four clotting time parameters: APTT, TT, PT, and PT-INR, all shortened with the increase of trimesters and gestational weeks and declined to the lowest value at the third trimester, and these changing trends are similar to the previous studies based on Chinese pregnant women. APTT mainly reflects the intrinsic coagulation pathway and is considered a good indicator for deficiencies of several copulation factors, including the only factor reduced during pregnancy: FXI. As a result of the large assumption of FXI, APTT may shorten as the trimesters and gestational weeks increase. Owing to the extrinsic coagulation pathway represented by PT, its shortening may reveal a decrease in the efficiency of synthesizing certain coagulation factors, such as FII, FV, FVII, and Fib, as these factors accumulate during pregnancy and reach their peak in the third trimester [17]. TT measures how long the Fib turns into fibrin. Previous studies reported shortened TT during pregnancy in Chinese pregnant women [4, 7, 17], while the change in TT among trimesters did not follow a specific trend in this study. As the following univariate and multivariate linear regression analysis showed that TT was negatively associated with gestational weeks, we think TT also steadily decreased during pregnancy in this study.

The level of Fib and D-dimer increased with the increase of trimesters and gestational weeks and reached their highest in the third trimester. These trends are in accordance with the established works based on the western and Chinese populations [4, 5, 17–19]. Fib, also known as clotting factor I, can be transformed by thrombin into a fibrin gel to form a clot, and thus its gestational evaluating trend in this study indicated the hypercoagulable state during pregnancy. Along with a localized increase in Fib formation, D-dimer progressively increased with the gestational age and trimesters due to its fibrinolytic activity sensitivity [20]. Also, the magnitude of the increasing D-dimer level with gestational weeks [multivariable coefficient: 0.081 (0.075 to 0.086, p < 0.001)] is more significant than all other parameters. We suggest this significant variation might be the main reason for using D-dimer to safely rule out pulmonary embolism (PE) and thus reduce the amount of unnecessary diagnostic imaging [21]. We endorse that the reference intervals and profiles of D-dimer in this study could facilitate the establishment of pregnancy-adapted scores or other methods for VTE screening for Chinese patients.

Overall, the variation of the five parameters during pregnancy based on the latest coagulation characteristics in Chinese pregnant women is comparable with established works, and the trimester-specific RIs were updated accordingly. The positive association of each parameter with gestational weeks is also comparable with the literature [7].

### The impact of advanced maternal age on gestational coagulation profiles

Recently, the maternal age at childbirth has continued to increase worldwide in China due to the adjustment of the childbirth policy. AMA has been considered one of the risk factors for thromboembolism, especially VTE, during pregnancy [22]. In 2006, a US cohort of 9,058,162 pregnancy admissions and 73,834 postpartum admissions showed that the risk of VTE was a 1.38-fold increase in AMA pregnant women [23]. Therefore, understanding the coagulation profiles of pregnant women at AMA could be helpful for the current and upcoming clinical practice in China.

As D-dimer showed a strong association with VTE in pregnancy, we expected that AMA might influence the gestational D-dimer level. However, consistent with an established study on Chinese pregnant women [24], D-dimer showed no statistical difference between the AMA and non-AMA groups. We also identified no association of D-dimer with age in univariate and multivariate linear regression analyses.

Although our investigations implied that the factor of AMA has little influence on the gestational D-dimer level, the impact on D-dimer during puerperium is unignorable. D-dimer > 5.50 mg/L might be an independent predictor of VTE in puerperium for Chinese AMA pregnant women [25].

Except for D-dimer, the investigation of the correlation of the other parameter with AMA is limited. Only a small sample study with 22 AMA US parturients undergoing elective cesarean delivery mentioned no significant difference in PT, PT-INR, APTT, and Fib between AMA and non-AMA groups before surgery [26]. Considering some underlying associations between thromboelastography (TEG) and the coagulation parameters [27], we also searched the impact of AMA on TEG and realized that the differences in gestational thromboelastographic profiles between AMA and non-AMA groups are not significant [26, 28]. These established studies imply that gestational coagulation profiles of healthy AMA and non-AMA women should be similar. In our study, no statistically significant difference in PT and Fib between AMA and non-AMA groups was identified, while significant differences in three other parameters: TT, PT-INR, and APPT, were observed. In multivariate linear regression analyses of the three parameters, only PT-INR [multivariate adjusted coefficient = 0.002 (0.000 to 0.003, p = 0.007) and APPT [multivariate adjusted coefficient = -0.003 (-0.005 to -0.001, p = 0.004)] showed significant association with age while the coefficients are small. This result implies that although differences in PT-INR and APPT between the two age groups are significant, the actual variation magnitude is small; For TT, its association with age was significant in univariate but not multivariate linear regression analyses, suggesting that this association was influenced by confounding factors and might not be genuine.

It is also worth noting that, based on several new studies indicating the possibility of progressive age-related risk for pregnancy over 35 years, the American College of Obstetricians and Gynecologists (ACOG) reported that dividing the age of individuals pregnant at 35 years using 5-year increments may help stratify the possible pregnancy risks associated with advancing age [29]. There, we encourage researchers to develop more comprehensive reference intervals for pregnancy over 35 years in the future in order to assess the potential for hypercoagulability better.

This study found statistically significant differences in several coagulation parameters between the AMA and non-AMA groups, even though the association between these parameters and age may be minor. Considering our findings and most previous research, AMA-specific reference intervals for Chinese pregnant women may be unnecessary in clinical practice.

### Exploring how obstetric history affects gestational coagulation parameter levels

Although parity and gravidity can influence the risks of the current pregnancy, research on how they affect gestational coagulation profiles is limited.

Fetal loss has generally been found to vary with gravidity, previous experience of fetal loss, and maternal age [30]. Women with higher gravidity should have a higher risk of fetal loss. Of all the coagulation factor deficiencies, only deficiency of FXIII, which can be preliminarily and roughly identified by PT and APTT [31], and Fib are associated with fetal loss [32]. As a result, factor XIII may decrease as gravity increases, and the shortened PT and PT-INR observed in this study may partially reveal this trend. However, APTT and Fib showed no clear variation trend, but D-dimer increased with increasing gravidity, which is difficult to explain as other risk factors for fetal loss could be confounders. We believe that determining the relationship between coagulation parameters and gravidity may necessitate the consideration of more independent variables, such as previous fetal loss experience and pregnancy outcome.

The risk of postpartum haemorrhage (PPH) has been reported to increase with parity, and a significant associating decrease in APTT was determined [33]. A decrease in Fib and an increase in D-dimer indicate the severity and onset of PPH [34–36]. Although the predictive and diagnostic value of PT in PPH was lack of reporting, it might be higher in patients with a higher risk of PPH due to the trend of extensive blood loss [35]. Overall, the lower Fib, higher D-dimer, and longer APTT, PT-INR observed in multiparous women may be associated with an increased risk of PPH. Some other studies might indicate the correlation between parity and coagulation profiles. Normal labor for primigravidae (gravidity 1 parity 0) showed a higher risk of developing preeclampsia than multigravida [37], and the decrease of Fib and prolonged TT might in the third trimester suggest the increased risk of preeclampsia [38]. However, the multivariable analyses in our study focused on the overall gestational period rather than the specific trimester, implying that additional research may be required trimester-specifically.

Furthermore, in a study that attempted to build maternal age/parity-specific RIs, the upper limit of the D-dimer RIs for parity 0 is lower than those for parity 1 (P < 0.05 when comparing groups with the same parity for the same age interval) [39], which is in agreement with the increasing association of parity 1 with D-dimer (vs. parity 0, p = 0.023) in our study. The decreased association of parity 1 with Fib, on the other hand, differed from the literature, and the other associations in our studies were not previously reported.

Overall, some of the associations discovered in this study may shed light on how varying parity and gravidity indicate the risk of fetal loss and PPH. More conclusions, however, cannot be drawn due to the lack of literature on the relationship between coagulation profiles and obstetric history during pregnancy.

### Limitation

The limitations of this study should be addressed. RIs in this study are only adaptable to clinical laboratories using the same reagent and coagulation analyzer. Previous pregnancy outcomes might also be crucial for the obstetric history and recommended being collected for future studies investigating the correlation between coagulation profiles and obstetric history. Moreover, we recommended including more pregnant women with pathological pregnancies, such as multiple or singleton pregnancies with preeclampsia or PPH, to further understand the diagnostic and screening potential of these gestational coagulation parameters.

## Conclusions

Owing to the potential changes in demographics and attitudes toward fertility following the change in childbirth policy in China, this work updated the coagulation profile of Chinese pregnant women with diverse obstetric histories and established the gestational trimester-specific RIs accordingly. No specific RIs based on AMA, gravidity, or parity might need to be set, though some association of coagulation parameters on parity and gravidity might indicate the risk of fetal loss and PPH. Overall, RIs established in this study should aid in the accurate and reliable monitoring of coagulation and fibrinolytic systems for the most recent pregnancy care and management in China.

## Electronic supplementary material

Below is the link to the electronic supplementary material.

**Additional file 1.** Supplementary tables

## Data Availability

All data generated or analyzed during this study are included in this article. Further enquiries can be directed to the corresponding authors.

## Abbreviations

RIs: Reference intervals
AMA: Advanced maternal age
VTE: Venous thromboembolism
PT: Prothrombin time
APTT: Activated partial thromboplastin time
TT: Thrombin time
Fib: Fibrinogen
IQR: Interquartile range
BMI: Body mass index
INR: International normalized ratio
SD: Standard deviation
TEG: Thromboelastography
PPH: Postpartum haemorrhage
