# Supplementary material for: Trimester-specific reference intervals and profile of coagulation parameters for Chinese pregnant women with diverse demographics and obstetric history: a cross-sectional study
Source: BMC Pregnancy Childbirth. 2023 Jun 6;23:421. doi: 10.1186/s12884-023-05571-z (PMC10245598; doi:10.1186/s12884-023-05571-z)
Supplement: Supplementary file 1 — Additional file 1. Supplementary tables [file 12884_2023_5571_MOESM1_ESM.docx]

**Table S1** Demographics, coagulation characteristics, and obstetric history of the pregnant women divided by the identification of AMA.

|  | | **< 35** | **≥ 35** | **p** |
| --- | --- | --- | --- | --- |
| Baseline BMI | Median  (IQR) | 20.761  (19.626-22.044) | 21.211  (19.942-22.656) |  |
|  | Mean  (SD) | 20.958  (1.613) | 21.320  (1.740) | 0.001 |
| Current BMI | Median  (IQR) | 23.415  (21.513-25.391) | 23.983  (22.210-25.926) |  |
|  | Mean  (SD) | 23.577  (2.768) | 24.135  (2.741) | 0.003 |
| APTT(s) | Median  (IQR) | 28.950  (27.300-30.900) | 28.600  (26.700-30.500) | 0.034 |
|  | Mean  (SD) | 29.172  (2.600) | 28.821  (2.769) |  |
| TT (s) | Median  (IQR) | 15.200  (14.900-15.700) | 15.400  (15.000-16.000) | < 0.01 |
|  | Mean  (SD) | 15.362  (0.854) | 15.514  (0.814) |  |
| PT | Median  (IQR) | 8.760  (8.468- 9.110) | 8.730  (8.400 -9.100) | 0.352 |
|  | Mean  (SD) | 8.800  (0.471) | 8.797  (0.535) |  |
| PT-INR | Median  (IQR) | 0.903  (0.875-0.938) | 0.916  (0.882-0.948) | 0.001 |
|  | Mean  (SD) | 0.907  (0.046) | 0.921  (0.055) |  |
| Fib (g/L) | Median  (IQR) | 4.070  (3.607- 4.502) | 4.080  (3.660- 4.600) | 0.221 |
|  | Mean  (SD) | 4.095  (0.665) | 4.150  (0.685) |  |
| D-dimer (µg/ml) | Median  (IQR) | 0.697  (0.378-1.185) | 0.747  (0.387-1.170) | 0.666 |
|  | Mean  (SD) | 0.922  (0.866) | 0.919  (0.790) |  |
| No. of pregnant women with  certain gravidity (%) | 1 | 312 (65.5) | 108 (26.0) | < 0.001 |
|  | 2 | 116 (24.4) | 132 (31.7) |  |
|  | 3 | 34 (7.1) | 89 (21.4) |  |
|  | > 3 | 14 (2.9) | 87 (20.9) |  |
| No. of pregnant women with  certain parity (%) | 0 | 396 (83.2) | 182 (43.8) | < 0.001 |
|  | 1 | 76 (16.0) | 219 (52.6) |  |
|  | 2 | 4 (0.8) | 14 (3.4) |  |
|  | 3 | 0 (0.0) | 1 (0.2) |  |

*Note:* BMI and all the coagulation parameters were skewed distributed, and the Kruskal-Wallis H test was used to test the difference of them among the groups; Differences in categorical data: No. of pregnant women with certain gravidity and parity were tested using Mantel-Hazel chi-square test.

**TABLE S2** Coagulation characteristics of the non-pregnant and pregnant women

|  | | **Non-pregnant women** | **Pregnant women** | **p** |
| --- | --- | --- | --- | --- |
| **APTT (s)** | Median (IQR) | 33.300 (30.50-35.80) | 28.700 (27.10-30.70) | < 0.01 |
|  | Mean (SD) | 33.562 (4.12) | 29.007 (2.68) |  |
| **TT (s)** | Median (IQR) | 16.300 (15.90-17.00) | 15.300 (14.90-15.80) | < 0.01 |
|  | Mean (SD) | 16.494 (0.92) | 15.434 (0.84) |  |
| **PT (s)** | Median (IQR) | 9.030 (8.71-9.40) | 8.740 (8.43-9.11) | < 0.01 |
|  | Mean (SD) | 9.048 (0.57) | 8.799 (0.50) |  |
| **PT-INR** | Median (IQR) | 0.950 (0.91-0.99) | 0.909 (0.88-0.94) | < 0.01 |
|  | Mean (SD) | 0.948 (0.06) | 0.914 (0.05) |  |
| **Fib (g/L)** | Median (IQR) | 2.780 (2.49-3.06) | 4.070 (3.64-4.57) | < 0.01 |
|  | Mean (SD) | 2.818 (0.46) | 4.11 (0.67) |  |
| **D-dimer (µg/ml)** | Median (IQR) | 0.163 (0.10-0.25) | 0.720 (0.38-1.17) | < 0.01 |
|  | Mean (SD) | 0.196 (0.13) | 0.920 (0.83) |  |

*Note: All the coagulation parameters were skewed distributed, and the Kruskal-Wallis H test was used to test the difference among groups.*

**Table S3** Coagulation reference intervals of healthy population regardless of gender.

|  | **Healthy population** |
| --- | --- |
|  |  |
| **APTT(s)** | 27.5-43.1 |
| **TT(s)** | 14.7-18.6 |
| **PT(s)** | 7.96-10.30 |
| **PT-INR** | 0.83-1.08 |
| **Fib (g/L)** | 2.00-3.89 |
| **D-dimer (µg/ml)** | <0.39 |
